# {6,6′-Dieth­oxy-2,2′-[ethyl­enebis(nitrilo­methyl­idyne)]diphenolato}nickel(II) monohydrate

**DOI:** 10.1107/S1600536808039822

**Published:** 2008-11-29

**Authors:** Hai Xie

**Affiliations:** a College of Chemistry & Chemical Engineering, Shanxi Datong University, Shanxi 037009, People’s Republic of China

## Abstract

In the title compound, [Ni(C_20_H_22_N_2_O_4_)]·H_2_O, the Ni^II^ ion and the water mol­ecule are located on a twofold rotation axis. The Ni ion is coordinated by two N [Ni—N = 1.8462 (18) Å] and two O [Ni—O = 1.8645 (14) Å] atoms in a distorted square-planar geometry. The water mol­ecule and the Ni complex mol­ecule are paired *via* O—H⋯O hydrogen bonds.

## Related literature

For details of the synthesis, see Mohanta *et al.* (2002 ▶). For a related crystal structure, see Yu (2006 ▶). For general background, see: Ghosh *et al.* (2006 ▶); Samanta *et al.* (2007 ▶); Singh *et al.* (2007 ▶); Yu *et al.* (2007 ▶).
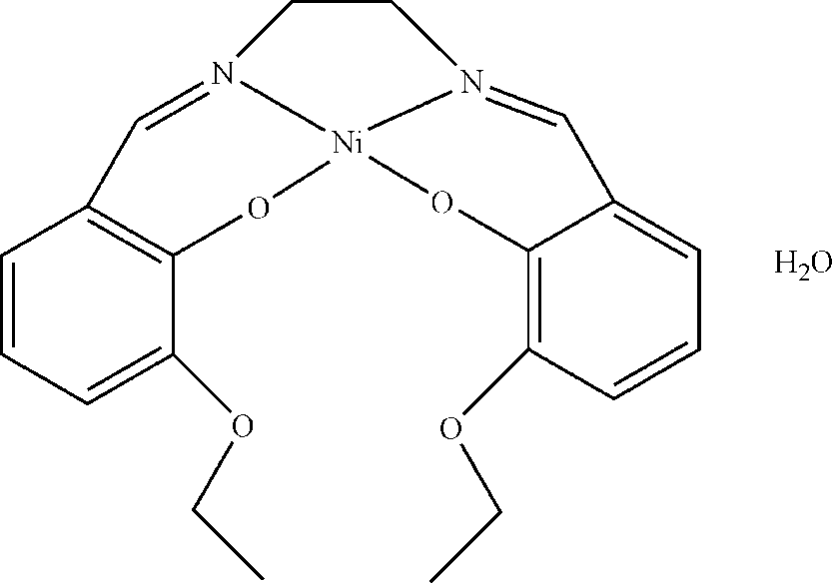


## Experimental

### 

#### Crystal data

[Ni(C_20_H_22_N_2_O_4_)]·H_2_O
*M* *_r_* = 431.12
Orthorhombic, 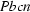

*a* = 12.8401 (8) Å
*b* = 19.6133 (12) Å
*c* = 7.5853 (5) Å
*V* = 1910.3 (2) Å^3^
*Z* = 4
Mo *K*α radiation
μ = 1.05 mm^−1^
*T* = 273 (2) K
0.15 × 0.13 × 0.11 mm

#### Data collection

Bruker APEXII CCD area-detector diffractometer
Absorption correction: multi-scan (*SADABS*; Sheldrick, 2003 ▶) *T*_min_ = 0.858, *T*_max_ = 0.893
8741 measured reflections
1676 independent reflections
1381 reflections with *I* > 2σ(*I*)
*R*_int_ = 0.025

#### Refinement

*R*[*F* ^2^ > 2σ(*F* ^2^)] = 0.027
*wR*(*F* ^2^) = 0.072
*S* = 1.04
1676 reflections
129 parameters
1 restraint
H-atom parameters constrained
Δρ_max_ = 0.33 e Å^−3^
Δρ_min_ = −0.41 e Å^−3^

### 

Data collection: *APEX2* (Bruker, 2004 ▶); cell refinement: *SAINT-Plus* (Bruker, 2001 ▶); data reduction: *SAINT-Plus*; program(s) used to solve structure: *SHELXS97* (Sheldrick, 2008 ▶); program(s) used to refine structure: *SHELXL97* (Sheldrick, 2008 ▶); molecular graphics: *XP* (Sheldrick, 1998 ▶); software used to prepare material for publication: *SHELXL97* (Sheldrick, 2008 ▶).

## Supplementary Material

Crystal structure: contains datablocks I, global. DOI: 10.1107/S1600536808039822/cv2487sup1.cif

Structure factors: contains datablocks I. DOI: 10.1107/S1600536808039822/cv2487Isup2.hkl

Additional supplementary materials:  crystallographic information; 3D view; checkCIF report

## Figures and Tables

**Table 1:** Hydrogen-bond geometry (Å, °)

*D*—H⋯*A*	*D*—H	H⋯*A*	*D*⋯*A*	*D*—H⋯*A*
O3—H3*A*⋯O2	0.82	2.10	2.8158 (15)	147

**Fig. 1.**
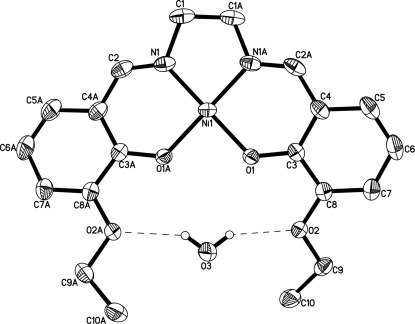
View of the title compound with the atom-labelling scheme [symmetry code: (A) -x + 1,y,-z + 1/2]. Displacement ellipsoids are drawn at the 30% probability level. Dashed lines denote H-bonds. C-bound H-atoms omitted for clarity.

## supplementary crystallographic information

### Comment

Schiff-bases have played an important role in the development of coordination chemistry as they readily form stable complexes with most of the transition metals, in which some could exhibit interesting properties (Yu *et al.*, 2007; Ghosh *et al.*, 2006; Singh *et al.*, 2007; Samanta *et al*., 2007). Herein we report a new Ni^II^ complex based on the tetradentate Schiff-base ligand *N*,*N*'-ethylenebis(3-ethoxysalicylaldimine).

The geometry and labeling scheme for the title compound are depicted in Figure 1. The coordination sphere for the Ni^II^ ion in the title complex is a slightly distorted square planar, in which the four positions are occupied by two N and two O atoms of the Schiff-base ligand. The mean deviation from the plane formed by the two N atoms, two O atoms and the Ni ion is only 0.025 Å. The bond lengths of Ni—N and Ni—O are 1.8462 (18) and 1.8645 (14) /A%, respectively, which are consistant with the corresponding distances in 6,6'-dimethoxy-2,2'-(ethane-1,2-diylbis(nitrilomethylidyne)diphenolato)- nickel(II) (Yu, 2006). The crystalline water molecule and Ni-complex are paired *via* O—H···.O hydrogen bonds (Table 1, Fig. 1).

### Experimental

The Schiff base ligand H_2_L (H_2_L= *N*,*N*'-ethylenebis(3-ethoxysalicylaldimine)) was prepared according to the reported method (Mohanta, *et al.*, 2002). The synthesis of the title complex was carried out by reacting Ni(CH_3_COO)_2_.4H_2_O, and H_2_L with the molar ratio 1:1 in methanol. After the stirring process was continued for about 30 min at room temperature, the mixture was filtered and the filtrate was allowed to partial evaporate in air for sevral days to produce crystals suitable for X-ray diffraction.

### Refinement

C-bound H atoms were placed in calculated positions, with C—H distances of 0.93 and 0.97 Å, respectively. Atom H3A was located on a difference Fourier map, but placed in idealized position with O—H = 0.82 Å. All H atoms were refined in riding model approximation, with *U*_iso_(H) = 1.2*U*_eq_(C, O).

### Crystal data

**Table:**

[Ni(C_20_H_22_N_2_O_4_)]·H_2_O	*F*_000_ = 904
*M**_r_* = 431.12	*D*_x_ = 1.499 Mg m^−^^3^
Orthorhombic, *P**b**c**n*	Mo *K*α radiation λ = 0.71073 Å
Hall symbol: -p 2n 2ab	Cell parameters from 2990 reflections
*a* = 12.8401 (8) Å	θ = 3.2–26.5º
*b* = 19.6133 (12) Å	µ = 1.05 mm^−^^1^
*c* = 7.5853 (5) Å	*T* = 273 (2) K
*V* = 1910.3 (2) Å^3^	Block, red-brown
*Z* = 4	0.15 × 0.13 × 0.11 mm

### Data collection

**Table:**

Bruker APEXII CCD area-detector diffractometer	1676 independent reflections
Radiation source: fine-focus sealed tube	1381 reflections with *I* > 2σ(*I*)
Monochromator: graphite	*R*_int_ = 0.025
*T* = 273(2) K	θ_max_ = 25.0º
φ and ω scans	θ_min_ = 1.9º
Absorption correction: multi-scan(SADABS; Sheldrick, 2003)	*h* = −15→14
*T*_min_ = 0.858, *T*_max_ = 0.893	*k* = −16→23
8741 measured reflections	*l* = −8→8

### Refinement

**Table:**

Refinement on *F*^2^	Secondary atom site location: difference Fourier map
Least-squares matrix: full	Hydrogen site location: inferred from neighbouring sites
*R*[*F*^2^ > 2σ(*F*^2^)] = 0.027	H-atom parameters constrained
*wR*(*F*^2^) = 0.072	*w* = 1/[σ^2^(*F*_o_^2^) + (0.0307*P*)^2^ + 0.9816*P*] where *P* = (*F*_o_^2^ + 2*F*_c_^2^)/3
*S* = 1.04	(Δ/σ)_max_ < 0.001
1676 reflections	Δρ_max_ = 0.33 e Å^−^^3^
129 parameters	Δρ_min_ = −0.41 e Å^−^^3^
1 restraint	Extinction correction: none
Primary atom site location: structure-invariant direct methods	

### Special details

**Table:**

Geometry. All e.s.d.'s (except the e.s.d. in the dihedral angle between two l.s. planes) are estimated using the full covariance matrix. The cell e.s.d.'s are taken into account individually in the estimation of e.s.d.'s in distances, angles and torsion angles; correlations between e.s.d.'s in cell parameters are only used when they are defined by crystal symmetry. An approximate (isotropic) treatment of cell e.s.d.'s is used for estimating e.s.d.'s involving l.s. planes.
Refinement. Refinement of *F*^2^ against ALL reflections. The weighted *R*-factor *wR* and goodness of fit *S* are based on *F*^2^, conventional *R*-factors *R* are based on *F*, with *F* set to zero for negative *F*^2^. The threshold expression of *F*^2^ > σ(*F*^2^) is used only for calculating *R*-factors(gt) *etc*. and is not relevant to the choice of reflections for refinement. *R*-factors based on *F*^2^ are statistically about twice as large as those based on *F*, and *R*- factors based on ALL data will be even larger.

### Fractional atomic coordinates and isotropic or equivalent isotropic displacement parameters (Å^2^)

**Table:**

	*x*	*y*	*z*	*U*_iso_*/*U*_eq_	
Ni1	0.5000	0.023585 (17)	0.2500	0.03740 (14)	
O1	0.41162 (10)	0.09308 (7)	0.17417 (19)	0.0406 (3)	
O2	0.32670 (11)	0.20336 (7)	0.03750 (19)	0.0454 (4)	
O3	0.5000	0.23474 (14)	0.2500	0.1240 (15)	
H3A	0.4599	0.2100	0.1962	0.149*	
N1	0.58936 (15)	−0.04538 (8)	0.3175 (2)	0.0457 (5)	
C1	0.54450 (19)	−0.11475 (10)	0.3139 (3)	0.0565 (6)	
H1A	0.5201	−0.1274	0.4305	0.068*	
H1B	0.5969	−0.1474	0.2773	0.068*	
C2	0.6841 (2)	−0.03851 (12)	0.3700 (3)	0.0546 (6)	
H2	0.7227	−0.0782	0.3842	0.066*	
C3	0.31834 (15)	0.08654 (10)	0.1050 (3)	0.0398 (5)	
C4	0.26463 (18)	0.02419 (11)	0.0914 (3)	0.0495 (6)	
C5	0.1623 (2)	0.02214 (15)	0.0224 (4)	0.0684 (8)	
H5	0.1269	−0.0192	0.0171	0.082*	
C6	0.1152 (2)	0.07964 (15)	−0.0361 (4)	0.0709 (8)	
H6	0.0474	0.0777	−0.0789	0.085*	
C7	0.16808 (17)	0.14183 (13)	−0.0323 (3)	0.0550 (6)	
H7	0.1361	0.1811	−0.0748	0.066*	
C8	0.26779 (16)	0.14498 (11)	0.0345 (3)	0.0427 (5)	
C9	0.28968 (19)	0.26143 (11)	−0.0578 (3)	0.0535 (6)	
H9A	0.2273	0.2793	−0.0024	0.064*	
H9B	0.2727	0.2486	−0.1780	0.064*	
C10	0.3732 (2)	0.31410 (12)	−0.0576 (4)	0.0677 (7)	
H10A	0.3886	0.3270	0.0616	0.102*	
H10B	0.3500	0.3534	−0.1223	0.102*	
H10C	0.4347	0.2959	−0.1120	0.102*	

### Atomic displacement parameters (Å^2^)

**Table:**

	*U*^11^	*U*^22^	*U*^33^	*U*^12^	*U*^13^	*U*^23^
Ni1	0.0425 (2)	0.0303 (2)	0.0394 (2)	0.000	0.01083 (17)	0.000
O1	0.0376 (8)	0.0359 (7)	0.0481 (8)	−0.0025 (6)	0.0007 (7)	−0.0011 (6)
O2	0.0452 (8)	0.0416 (8)	0.0493 (9)	0.0033 (7)	−0.0080 (7)	0.0010 (7)
O3	0.132 (3)	0.0510 (17)	0.189 (4)	0.000	−0.113 (3)	0.000
N1	0.0557 (12)	0.0352 (9)	0.0464 (10)	0.0072 (9)	0.0186 (9)	0.0028 (8)
C1	0.0755 (17)	0.0324 (11)	0.0617 (16)	0.0066 (11)	0.0279 (12)	0.0043 (10)
C2	0.0599 (16)	0.0458 (14)	0.0581 (15)	0.0219 (12)	0.0157 (12)	0.0051 (11)
C3	0.0369 (11)	0.0475 (12)	0.0350 (11)	−0.0042 (9)	0.0075 (9)	−0.0053 (9)
C4	0.0469 (13)	0.0523 (13)	0.0492 (13)	−0.0144 (11)	0.0076 (11)	−0.0052 (11)
C5	0.0511 (15)	0.0715 (18)	0.082 (2)	−0.0239 (13)	0.0014 (14)	−0.0063 (15)
C6	0.0411 (14)	0.092 (2)	0.0792 (19)	−0.0118 (14)	−0.0080 (13)	−0.0123 (17)
C7	0.0427 (13)	0.0687 (16)	0.0538 (14)	0.0038 (12)	−0.0028 (11)	−0.0060 (12)
C8	0.0392 (12)	0.0522 (13)	0.0367 (11)	−0.0006 (10)	0.0034 (9)	−0.0073 (10)
C9	0.0616 (15)	0.0533 (14)	0.0455 (13)	0.0159 (12)	−0.0076 (11)	−0.0005 (11)
C10	0.0848 (19)	0.0510 (14)	0.0673 (17)	0.0039 (14)	−0.0112 (15)	0.0131 (13)

### Geometric parameters (Å, °)

**Table:**

Ni1—N1^i^	1.8462 (18)	C3—C8	1.422 (3)
Ni1—N1	1.8462 (18)	C4—C5	1.415 (3)
Ni1—O1^i^	1.8645 (14)	C4—C2^i^	1.426 (3)
Ni1—O1	1.8645 (14)	C5—C6	1.354 (4)
O1—C3	1.314 (2)	C5—H5	0.9300
O2—C8	1.372 (2)	C6—C7	1.396 (3)
O2—C9	1.431 (2)	C6—H6	0.9300
O3—H3A	0.8168	C7—C8	1.378 (3)
N1—C2	1.287 (3)	C7—H7	0.9300
N1—C1	1.478 (3)	C9—C10	1.489 (3)
C1—C1^i^	1.498 (5)	C9—H9A	0.9700
C1—H1A	0.9700	C9—H9B	0.9700
C1—H1B	0.9700	C10—H10A	0.9600
C2—C4^i^	1.426 (3)	C10—H10B	0.9600
C2—H2	0.9300	C10—H10C	0.9600
C3—C4	1.408 (3)		
			
N1^i^—Ni1—N1	85.77 (12)	C5—C4—C2^i^	118.7 (2)
N1^i^—Ni1—O1^i^	178.06 (7)	C6—C5—C4	120.8 (2)
N1—Ni1—O1^i^	94.12 (7)	C6—C5—H5	119.6
N1^i^—Ni1—O1	94.12 (7)	C4—C5—H5	119.6
N1—Ni1—O1	178.06 (7)	C5—C6—C7	120.2 (2)
O1^i^—Ni1—O1	86.06 (8)	C5—C6—H6	119.9
C3—O1—Ni1	127.35 (13)	C7—C6—H6	119.9
C8—O2—C9	118.21 (16)	C8—C7—C6	119.9 (2)
C2—N1—C1	118.07 (19)	C8—C7—H7	120.1
C2—N1—Ni1	126.62 (16)	C6—C7—H7	120.1
C1—N1—Ni1	115.29 (16)	O2—C8—C7	123.7 (2)
N1—C1—C1^i^	108.01 (14)	O2—C8—C3	114.51 (17)
N1—C1—H1A	110.1	C7—C8—C3	121.8 (2)
C1^i^—C1—H1A	110.1	O2—C9—C10	108.22 (18)
N1—C1—H1B	110.1	O2—C9—H9A	110.1
C1^i^—C1—H1B	110.1	C10—C9—H9A	110.1
H1A—C1—H1B	108.4	O2—C9—H9B	110.1
N1—C2—C4^i^	126.2 (2)	C10—C9—H9B	110.1
N1—C2—H2	116.9	H9A—C9—H9B	108.4
C4^i^—C2—H2	116.9	C9—C10—H10A	109.5
O1—C3—C4	124.1 (2)	C9—C10—H10B	109.5
O1—C3—C8	119.19 (18)	H10A—C10—H10B	109.5
C4—C3—C8	116.69 (19)	C9—C10—H10C	109.5
C3—C4—C5	120.4 (2)	H10A—C10—H10C	109.5
C3—C4—C2^i^	120.5 (2)	H10B—C10—H10C	109.5

Symmetry codes: (i) −*x*+1, *y*, −*z*+1/2.

### Hydrogen-bond geometry (Å, °)

**Table:**

*D*—H···*A*	*D*—H	H···*A*	*D*···*A*	*D*—H···*A*
O3—H3A···O1	0.82	2.38	3.056 (3)	140
O3—H3A···O2	0.82	2.10	2.8158 (15)	147
